# Preserve encephalus in surgery of trauma: online survey. (P.E.S.T.O)

**DOI:** 10.1186/s13017-019-0229-2

**Published:** 2019-03-04

**Authors:** Edoardo Picetti, Ronald V. Maier, Sandra Rossi, Andrew W. Kirkpatrick, Walter L. Biffl, Philip F. Stahel, Ernest E. Moore, Yoram Kluger, Gian Luca Baiocchi, Luca Ansaloni, Vanni Agnoletti, Fausto Catena

**Affiliations:** 1 Department of Anesthesia and Intensive Care, Parma University Hospital, Via Gramsci 14, 43100 Parma, Italy; 2 Department of Surgery, Harborview Medical Center, Seattle, USA; 3 Departments of General Acute Care, Abdominal Wall Reconstruction and Trauma Surgery, Foothills Medical Centre, Calgary, Canada; 4 Division of Trauma and Acute Care Surgery, Scripps Memorial Hospital, La Jolla, California, USA; 5 College of Osteopathic Medicine, Rocky Vista University, Parker, CO USA; 6 Department of Trauma Surgery, Denver Health, Denver, CO USA; 7 Department of General Surgery, Rambam Health Campus, Haifa, Israel; 8 Department of Clinical and Experimental Sciences, University of Brescia, Brescia, Italy; 9 Department of General and Emergency Surgery, Bufalini Hospital, Cesena, Italy; 10 Department of Anesthesia and Intensive Care, Bufalini Hospital, Cesena, Italy; 11 Department of Emergency Surgery, Parma University Hospital, Parma, Italy

**Keywords:** Traumatic brain injury, Polytrauma, Management, Monitoring

## Abstract

**Background:**

Traumatic brain injury (TBI) is a global health problem. Extracranial hemorrhagic lesions needing emergency surgery adversely affect the outcome of TBI. We conducted an international survey regarding the acute phase management practices in TBI polytrauma patients.

**Methods:**

A questionnaire was available on the World Society of Emergency Surgery website between December 2017 and February 2018. The main endpoints were the evaluation of (1) intracranial pressure (ICP) monitoring during extracranial emergency surgery (EES), (2) hemodynamic management without ICP monitoring during EES, (3) coagulation management, and (4) utilization of simultaneous multisystem surgery (SMS).

**Results:**

The respondents were 122 representing 105 trauma centers worldwide. ICP monitoring was utilized in 10–30% of patients at risk of intracranial hypertension (IH) undergoing EES from about a third of the respondents [*n* = 35 (29%)]. The respondents reported that the safest values of systolic blood pressure during EES in patients at risk of IH were 90–100 mmHg [*n* = 35 (29%)] and 100–110 mmHg [*n* = 35 (29%)]. The safest values of mean arterial pressure during EES in patients at risk of IH were > 70 mmHg [*n* = 44 (36%)] and > 80 mmHg [*n* = 32 (26%)]. Regarding ICP placement, a large percentage of respondents considered a platelet (PLT) count > 50,000/mm^3^ [*n* = 57 (47%)] and a prothrombin time (PT)/activated partial thromboplastin time (aPTT) < 1.5 times the normal control [*n* = 73 (60%)] to be the safest parameters. For craniotomy, the majority of respondents considered PLT count > 100,000/mm^3^ [*n* = 67 (55%)] and a PT/aPTT < 1.5 times the normal control [*n* = 76 (62%)] to be the safest parameters. Almost half of the respondents [*n* = 53 (43%)], reported that they transfused red blood cells (RBCs)/plasma (P)/PLTs at a ratio of 1/1/1 in TBI polytrauma patients. SMS was performed in 5–19% of patients, requiring both an emergency neurosurgical operation and EES, by almost half of the respondents [*n* = 49 (40%)].

**Conclusions:**

A great variability in practices during the acute phase management of polytrauma patients with severe TBI was identified. These findings may be helpful for future investigations and educational purposes.

**Electronic supplementary material:**

The online version of this article (10.1186/s13017-019-0229-2) contains supplementary material, which is available to authorized users.

## Background

Traumatic brain injury (TBI) is a leading cause of mortality and disability worldwide, with devastating impact on patients and their families [1]. Systemic hemorrhage represents the leading cause of preventable death among trauma patients [2, 3], and in polytrauma patients, TBI is frequently associated with extracranial hemorrhage that is known to worsen outcome by exacerbating secondary insults (e.g., intracranial hypertension and arterial hypotension with cerebral hypoperfusion) [7]. Clinical strategies, including damage control resuscitation (DCR) and damage control surgery (DCS), play a key role in the management of hemorrhage in trauma patients [4–6]. DCS techniques include (1) rapid control of hemorrhage and contamination, (2) temporary wound closure, (3) resuscitation to normal physiological parameters in the intensive care unit (ICU), and (4) subsequent re-exploration and definitive repair following restoration of normal physiological variables [5]. DCR represents a nonsurgical strategy, routinely associated with DCS, consisting of (1) permissive hypotension to reduce bleeding, (2) minimal use of crystalloid fluids, and (3) utilization of blood and blood products to improve hemostasis [4, 6]. Unfortunately, little is known concerning the acute optimal monitoring and management strategies in this setting. For these reasons, we conducted an international survey regarding the acute phase management practices in polytrauma patients with severe TBI.

## Methods

This survey was promoted and endorsed by the World Society of Emergency Surgery (WSES). The questionnaire was composed of 24 items and available on the WSES website and associated newsletters (https://docs.google.com/forms/d/1Y7-L1ZNJWIlb9e4ea9QQkGqQZYZ2Byr2uNcHXZI4nxI/edit—Additional file 1: Appendix 1) from December 1, 2017 through February 28, 2018. The survey was developed by three investigators (E.P., S.R., and F.C.) following a nonsystematic review of the literature concerning acute phase management practices in polytrauma patients with severe TBI. Specific questions were formulated to target selective important issues surrounding this topic. The main endpoints of the survey were the evaluation of (1) intracranial pressure (ICP) monitoring during extracranial emergency surgery (EES), (2) hemodynamic management without ICP monitoring during EES, (3) coagulation management, and (4) utilization of simultaneous multisystem surgery (SMS) techniques. Moreover, we endeavored to assess whether a relationship exists between the center admission volume and the adherence to existing guidelines and clinical protocols. Our hypothesis was that a high compliance with existing guidelines and protocols would be expected in trauma centers with large admission rates. The target audience was emergency surgeons who routinely treat polytrauma patients with TBI. Moreover, the questionnaire investigators invited the target participants to involve additional respondents locally. Participants did not receive compensation for their participation in the survey; all those who agreed are identified as contributors at the end of the manuscript. No ethical approval was required.

### Statistical analysis

Data from the questionnaire were retrieved from the website database and subsequently stored as an Excel file (Microsoft Corp, Redmond, WA). Descriptive statistics were computed for all study variables. The results are presented as numbers (percentage of the total respondents). To analyze the relationship between the volume of major trauma admission [Injury Severity Score (ISS) > 15] per year and the rates of compliance with guidelines and established protocols, centers were divided in two group: (1) group A: < 250 major trauma admissions/year and (2) group B: ≥ 250 major trauma admissions/year. The following protocols were considered in compliance with the current guidelines [8, 9]: maintenance of systolic blood pressure (SBP) >  100 mmHg for patients at risk of intracranial hypertension (IH) during EES, maintenance of mean arterial pressure (MAP) > 80 mmHg for patients at risk of IH during EES, a platelet (PLT) count > 100,000/mm^3^ for ICP placement and craniotomy, a prothrombin time (PT)/activated partial thromboplastin time (aPTT) < 1.5 to normal control for ICP placement and craniotomy, and a red blood cell (RBC)/plasma (P)/PLT count at a ratio of 1 or 2/1/1 for DCR in patients with polytrauma and polytrauma with TBI. The presence of protocols was considered for ICP monitoring and use of SMS. Data were ordered in 2 × 2 contingency tables, and chi-square test or Fischer exact test were utilized for statistical analysis with Bonferroni correction due to multiple comparisons. Stata software release 13.0 was used for data analysis (StataCorp, 2013, Stata Statistical Software, Release 13; StataCorp LP, College Station, TX).

## Results

The number of respondents of the survey was 122 from 105 centers worldwide. The majority of respondents (*n* = 70 [57%]) were from Europe. When respondents were stratified by country, Italy was the country with the highest number of respondents (*n* = 22), followed by the USA (*n* = 18), Greece (*n* = 8), and Germany (*n* = 6) (Additional file 1: Table S1). Baseline characteristics of the survey participants are shown in Table 1. The majority of respondents are employed at a level I trauma center (*n* = 66 [54%]). No statistically significant relationship was observed between the volume of major trauma admissions and compliance rates with guidelines and established protocols (Additional file 1: Table S2)

**Table 1:** Baseline characteristics of the population

	Respondents (*n* = 122)
Years of practice in emergency surgery	
< 5	14 (12%)
6–10	32 (26%)
11–15	24 (20%)
16–20	15 (12%)
21–25	15 (12%)
> 25	22 (18%)
Trauma center level	
I	66 (54%)
II	37 (30%)
III	19 (16%)
Trauma team leader	
*Emergency surgeon*	78 (75%)
*Anesthesiologist*	16 (13%)
*Emergency medicine physician*	10 (8%)
*ICU physician*	5 (4%)
*Trauma team not present*	9 (7%)
*Other*	4 (3%)
Admission of trauma patients with ISS > 15	
< 250	44 (36%)
250–500	51 (42%)
500–750	14 (12%)
750–1000	4 (3%)
> 1000	9 (7%)
Admission of trauma patients with ISS > 15 and severe TBI (GCS < 8)	
< 100	53 (44%)
100–200	43 (35%)
200–300	15 (12%)
300–400	7 (6%)
> 400	4 (3%)
Performance of neurosurgical intervention during training in emergency surgery	
*Yes*	27 (22%)
*No*	95 (78%)

*Abbreviations*: *ICU* intensive care unit, *ISS* injury severity score, *GCS* Glasgow Coma Scale, *TBI* traumatic brain injury

### ICP monitoring during EES (Table 2)

ICP monitoring was utilized from about a third of respondents (*n* = 35, 29%) in 10–30% of patients who were determined to be at risk for IH undergoing EES (immediately after admission). ICP probes were inserted almost exclusively by neurosurgeons [*n* = 117 (96%)]. Although only a portion of respondents utilized an ICP monitoring protocol in this setting [*n* = 48 (39%)], 102 (84%) respondents considered the use of ICP monitoring during EES important, very important, or mandatory. In polytrauma patients with ICP monitoring and IH, extracranial pressures (such as intrathoracic pressure and abdominal pressure) were monitored by the majority of respondents [*n* = 93 (76%)].

**Table 2:** ICP monitoring during EES

	Respondents (*n* = 122)
ICP insertion	
*Neurosurgeon* (*attending*)	47 (39%)
*Neurosurgeon* (*resident*)	38 (31%)
*Neurosurgeon* (*attending* + *resident*)	32 (26%)
*Emergency surgeon* (*attending*)	1 (1%)
*Emergency surgeon* (*attending*) *+ neurosurgeon* (*attending*)	1 (1%)
*Other*	3 (2%)
ICP monitoring in patients (%) at risk of IH during EES (immediately after admission)	
0%	15 (13%)
< 10%	21 (17%)
10–30%	35 (29%)
30–50%	4 (3%)
50–70%	15 (12%)
70–99%	23 (19%)
100%	9 (7%)
Protocol for ICP monitoring in patients at risk of IH during EES (immediately after admission)	
*Yes*	48 (39%)
*No*	74 (61%)
Importance of ICP monitoring in patients at risk of IH during EES (immediately after admission)	
*Not important*	1 (1%)
*Somewhat important*	19 (15%)
*Important*	40 (33%)
*Very important*	46 (38%)
*Mandatory*	16 (13%)

*Abbreviations*: *ICP* intracranial pressure, *IH* intracranial hypertension, *EES* emergency extracranial surgery

### Hemodynamic management without ICP monitoring during EES (Table 3)

The respondents reported that the safest values of SBP during EES in patients at risk of IH were 90–100 mmHg [*n* = 35 (29%)] and 100–110 mmHg [*n* = 35 (29%)]. The safest values of MAP during EES in patients at risk of IH were > 70 mmHg [*n* = 44 (36%)] and > 80 mmHg [*n* = 32 (26%)].

**Table 3:** Hemodynamic management without ICP monitoring during EES

	Respondents (*n* = 122)
Safe SBP in patients at risk of IH during EES	
< 70 mmHg	1 (1%)
70–80 mmHg	10 (8%)
80–90 mmHg	20 (16%)
90–100 mmHg	35 (29%)
100–110 mmHg	35 (29%)
> 110 mmHg	21 (17%)
Safe MAP in patients at risk of IH during EES	
> 60 mmHg	24 (20%)
> 70 mmHg	44 (36%)
> 80 mmHg	32 (26%)
> 90 mmHg	22 (18%)

*Abbreviations*: *SBP* systolic blood pressure, *MAP* mean arterial pressure, *EES* emergency extracranial surgery

### Coagulation management (Table 4)

Regarding ICP placement, a large percentage of respondents considered a safe PLT count to be > 50,000/mm^3^ [*n* = 57 (47%)] and a PT/aPTT < 1.5 times the normal control [*n* = 73 (60%)]. For craniotomy, the majority of respondents considered a safe PLT value to be > 100,000/mm^3^ [*n* = 67 (55%)] and a PT/aPTT < 1.5 times the normal control [*n* = 76 (62%)]. A large percentage of respondents [*n* = 51 (42%)], in polytrauma patients during DCR, routinely transfused RBCs/P/PLTs at a ratio of 1/1/1. This strategy was utilized [*n* = 53 (43%)] also in polytrauma patients with TBI undergoing DCR.

**Table 4:** Coagulation management

Safe platelet count	ICP placement	Craniotomy
> 50,000 mm^3^	57 (47%)	44 (36%)
> 100,000 mm^3^	56 (46%)	67 (55%)
> 150,000 mm^3^	9 (7%)	11 (9%)
PT/aPTT	ICP placement	Craniotomy
1.2 times the normal control	43 (35%)	41 (34%)
1.5 times the normal control	73 (60%)	76 (62%)
1.8 times the normal control	6 (5%)	5 (4%)
RBCs/P/PLTs	DCR polytrauma	DCR polytrauma with TBI
1 RBC/1 P/1 PLT	51 (42%)	53 (43%)
2 RBCs/1 P/1 PLT	42 (34%)	40 (33%)
3 RBCs/1 P/1 PLT	20 (16%)	19 (16%)
Other	9 (8%)	10 (8%)

*Abbreviations*: *ICP* intracranial pressure, *PT* prothrombin time, *aPTT* activated partial thromboplastin time, *RBC* red blood cell, *P* plasma, *PLT* platelet, *DCR* damage control resuscitation, *TBI* traumatic brain injury

### Utilization of SMS (Table 5)

The results of our survey revealed that SMS is routinely performed in 5–19% of patients who require both an emergency neurosurgical operation and EES by 40% of the respondents (*n* = 49). Rather surprisingly, a strikingly low percentage of respondents reported that they routinely utilize a protocol for SMS [*n* = 33 (27%)]. However, most of the investigators who responded (*n* = 112 (92%) considered the ability to perform SMS important, very important, or mandatory.

**Table 5:** Utilization of SMS

	Respondents (*n* = 122)
Percentage of patients needing SMS (intracranial + extracranial^a^) and effectively submitted to SMS in acute care setting	
0%	17 (14%)
< 5%	13 (11%)
5–19%	49 (40%)
20–39%	22 (18%)
40–59%	7 (6%)
60–99%	8 (7%)
100%	6 (4%)
Protocol for SMS (intracranial + extracranial^a^) in acute care setting	
*Yes*	33 (27%)
*No*	89 (73%)
Importance of the ability to perform SMS (intracranial + extracranial^a^) in acute care setting	
*Not important*	0 (0%)
*Somewhat important*	10 (8%)
*Important*	40 (33%)
*Very important*	53 (43%)
*Mandatory*	19 (16%)

^a^Including radiologic interventional procedures

*Abbreviations*: *SMS* simultaneous multisystem surgery

## Discussion

This international survey provides important information regarding worldwide acute phase management practices in polytrauma TBI patients with particular focus on (1) ICP monitoring during EES, (2) hemodynamic management without ICP monitoring during EES, (3) coagulation management, and (4) utilization of SMS techniques.

### ICP monitoring during EES

IH is a dangerous secondary insult for the injured brain and is known to be associated with increased mortality and disability [10–12]. Invasive ICP monitoring can be considered a milestone in the management of TBI, allowing caregivers to provide therapies in an appropriate and timely manner. Moreover, the use of ICP monitoring has become useful for the estimation and management of cerebral perfusion pressure (CPP) [13, 14]. To this end, the most recent Brain Trauma Foundation (BTF) guidelines for TBI [8], regarding ICP monitoring, state that “Management of severe TBI patients using information from ICP monitoring is recommended to reduce in-hospital and 2-week post-injury mortality” (level IIB). Unfortunately, specific indications regarding which patients require ICP monitoring have not been determined or supported by evidence-based data [15]. However, the results of two recent consensus conferences [16, 17], written after the publication of the Benchmark Evidence From South American Trials: Treatment of Intracranial Pressure trial [18], recommend that [1] ICP should be monitored in all salvageable comatose patients with radiological signs of IH, while [2] ICP monitoring is not required in patients with minimal intracranial pathology (such as diffuse axonal injury or small petechiae). In one of these publications [16], ICP monitoring is recommended for TBI comatose patients with brain contusions in whom the interruption of sedation to check the neurological status is considered dangerous (e.g., instances of radiological signs of IH, severe respiratory failure, or ongoing EES). This latter recommendation remains clinically important since IH can complicate extracranial surgery performed within 2 weeks of trauma in severe/moderate TBI. [19, 20]. Kinoshita and colleagues [21] performed a small retrospective observational study to explore outcomes of polytrauma patients who underwent concurrent bleeding control and ICP monitoring using a specially designed hybrid emergency room system (a trauma resuscitation room that is equipped for the completion of all examinations and treatments in a single place). These authors demonstrated that this approach is feasible and should be evaluated in a larger, prospective, and interventional study. Despite the fact that most of our survey respondents considered ICP monitoring either important, very important, or mandatory during EES, it is routinely utilized in very few cases. Moreover, protocols for ICP monitoring during EES have not been utilized for the majority of respondents. These findings suggest that educational programs should be developed by organized medical societies and distributed worldwide, with the aim of increasing the prevalence of this type of monitoring during EES in patients at risk of IH. Furthermore, additional studies are warranted since data regarding EES, immediately after admission, remain primarily unavailable.

### Hemodynamic management without ICP monitoring during EES

Arterial hypotension, with associated cerebral hypoperfusion, is a frequently observed secondary insult during extracranial surgery in TBI [19, 20]. Decreased CPP, as well as elevated ICP, are associated with unfavorable neurological outcome after TBI [22]. Episodes of low CPP therefore need to be diagnosed and rapidly treated. Recent BTF guidelines [8] recommend maintenance of CPP between 60 and 70 mmHg. To obtain CPP values in this range, the utilization of ICP and MAP monitoring (CPP = MAP−ICP) is required [13]. In the absence of ICP monitoring, BTF guidelines [8] recommend the maintenance of SBP at 100 mmHg for patients 50–69 years old or 110 mmHg or above for patients 15–49 or > 70 years old. The most recent European guidelines regarding the management of major hemorrhage and coagulopathy in polytrauma patients with severe TBI [9] recommend the maintenance of MAP ≥ 80 mmHg until hemorrhage has ceased (grade 1C). Interestingly, regarding the requirement to maintain SBP during EES in patients at risk of IH without the availability of ICP monitoring, the majority of respondents (66–54%) considered values < 100 mmHg to be safe, regardless of the established BTF recommendations [13]. Moreover, regarding MAP during EES in patients at risk of IH without available ICP monitoring, a respectable percentage of respondents (54–44%) considered safe values to be > 80 mmHg according to European recommendations [9]. Probably, the choice of our respondents could be influenced by the increase in worldwide utilization of DCR protocols in polytrauma patients. However, targeted parameters for maintenance of blood pressure should be higher in polytrauma patients with TBI. These data suggest that additional educational efforts are required to increase clinical awareness concerning established and published recommendations with the aim to improve outcome in TBI polytrauma patients.

### Coagulation management

Coagulopathy is frequently encountered after trauma and, if not treated, is associated with increased mortality [23]. In polytrauma patients with TBI, coagulopathy is known to be associated also with further progression of IH and unfavorable neurological outcome [24, 25]. The most recent European guideline concerning the management of major hemorrhage and coagulopathy following trauma [9] recommended that PT and aPTT be maintained < 1.5 times the normal control (grade 1C) and the PLT count be maintained above 50,000/mm^3^ (grade 1C). Maintenance of a PLT count > 100,000/mm^3^ was also recommended for patients with ongoing bleeding and/or TBI (grade 2C). To our knowledge, no specific guidelines regarding coagulation management in TBI patients have been published, to date. Regarding conformity with ICP placement and craniotomy, the survey’s responses are in accordance with the above mentioned guidelines [9]. However, we believe that ICP placement, as a neurosurgical procedure, should be performed under conditions of a PLT count > 100,000/mm^3^. With respect to the RBC/P/PLT, the preference of the majority of our respondents (1 RBC/1 P/1 PLT in DCR with and without TBI) is in agreement with current data showing an improvement in outcomes with a ratio of 1/1/1 [26, 27].

### Utilization of SMS

Exsanguinating polytrauma TBI patients often require simultaneous operative management performed by different surgical teams [28–30]. The principal objective is cessation of hemorrhage and the prevention of secondary brain insults. This approach, which requires established protocols and a strict collaboration between different surgical teams (including interventional radiologists), is frequently utilized in military warfare situations but rarely in the civilian setting [28]. Kinoshita and colleagues [29] performed a retrospective study to evaluate the effects of a hybrid emergency room (ER) on functional outcomes in polytrauma TBI patients. This system facilitates the performance of diagnostic procedures [e.g., ultrasonography, radiography, computed tomography (CT)], as well as the application of damage control interventions (e.g., surgery, transarterial embolization, burr-hole craniotomy) simultaneously without patient transfer. These authors reported that the use of the hybrid ER system was significantly associated with both shorter times to initiate CT scanning/emergency surgery and fewer unfavorable outcomes at 6 months post injury. The results of our survey demonstrate that relatively few respondents are equipped to perform SMS in patients needing both an emergency neurosurgical operation and EES. Moreover, protocols and training programs are strikingly lacking but considered mandatory in order to synchronize and optimize the activities of different trauma specialists. The vast majority of respondents (> 90%) considered the ability to perform SMS important, very important, or mandatory. The results of our survey suggest that these concepts should be adopted by international medical societies. Further investigation into the utility of SMS in polytrauma TBI patients is warranted.

### Limitations

The creation and results of the survey described in the present study are associated with several limitations, including the relatively low number of respondents and the inability to calculate response rates based on the survey design. However, we obtained responses from 105 centers worldwide with vastly different resources, enhancing the generalizability of our observations. Moreover, in order to make the survey more accessible and straightforward in design, we chose to focus on specific topics to the exclusion of other equally important questions. In this regard, viscoelastic testing [i.e., thromboelastography (TEG), rotational thromboelastometry (ROTEM)] are increasingly used and are very useful considering the frequent administration of the novel oral anticoagulants (NOACs) [9, 31–33].

## Conclusions

A great variability in worldwide clinical practices for acute phase management of severe TBI patients with polytrauma was identified from the results of our survey. These novel observations will be helpful to define future investigations on this topic and underscore the need for further research efforts for optimized protocol-driven care in this important area.

## Additional file

Additional file 1: Appendix 1 Questionnaire. **Table S1.** The countries of responders. **Table S2.** Relationship between the volume of major trauma admissions and the compliance with guidelines and the presence of protocols. (DOCX 30 kb)

## Abbreviations

aPTT: Activated partial thromboplastin time
BTF: Brain Trauma Foundation
CPP: Cerebral perfusion pressure
CT: Computed tomography
DCR: Damage control resuscitation
DCS: Damage control surgery
EES: Emergency extracranial surgery
ER: Emergency room
ICU: Intensive care unit
IH: Intracranial hypertension
ISS: Injury Severity Score
MAP: Mean arterial pressure
NOAC: Novel oral anticoagulant
P: Plasma
PLT: Platelet
PT: Prothrombin time
RBC: Red blood cell
ROTEM: Rotational thromboelastometry
SBP: Systolic blood pressure
SMS: Simultaneous multisystem surgery
TBI: Traumatic brain injury
TEG: Thromboelastography
WSES: World Society of Emergency Surgery
