# Supplementary material for: Preserve encephalus in surgery of trauma: online survey. (P.E.S.T.O)
Source: World J Emerg Surg. 2019 Mar 4;14:9. doi: 10.1186/s13017-019-0229-2 (PMC6399949; doi:10.1186/s13017-019-0229-2)
Supplement: Supplementary file 1 — Appendix 1 Questionnaire. Table S1. The countries of responders. Table S2. Relationship between the volume of major trauma admissions and the compliance with guidelines and the presence of protocols. (DOCX 30 kb) [file 13017_2019_229_MOESM1_ESM.docx]

**Additional file 1: Appendix 1. Questionnaire.**

**1.** In which country do you practice?

**2.** Numbers of years of practice in emergency surgery (acute care surgery + trauma):

- < 5

- 6-10

- 11-15

- 16-20

- 21-25

- > 25

**3.** During your training in emergency surgery did you perform neurosurgical interventions?

- Yes

- No

**4.** Trauma center level:

- I

- II

- III

**5.** Annual number of major trauma admissions [Injury Severity Score (ISS) > 15]:

- < 250

- 250 - 500

- 500 - 750

- 750 - 1000

- > 1000

**6.** Annual number of major trauma patient admissions with associate severe traumatic brain injury (TBI) [Glasgow Coma Scale (GCS) < 8]:

- < 100

- 100 - 200

- 200 - 300

- 300 - 400

- > 400

**7.** At you center, generally who is the trauma team leader?

- anesthesiologist

- Intensive Care Unit (ICU) physician

- emergency surgeon

- emergency medicine physician

- trauma team not present

- other (please specify)

**8.** At your center, usually who insert intracranial pressure (ICP) probe in TBI with polytrauma in emergency setting? (more than one answer permitted)

- neurosurgeon (resident)

- neurosurgeon (attending)

- emergency surgeon (resident)

- emergency surgeon (attending)

- other (please specify)

**9.** At your center, usually who perform emergency trauma craniotomies in polytrauma patients? (more than one answer permitted)

- neurosurgeon

- emergency surgeon

- other (please specify)

**10.** At you center, what is the percentage of patients at risk of intracranial hypertension undergoing emergency extracranial surgery* (immediately after admission) has ICP monitoring?

**11.** At you center, do you have a protocol to standardize ICP monitoring in patients at risk for intracranial hypertension undergoing emergency extracranial surgery* ?

- Yes

- No

**12.** In your daily clinical practice, do you consider the placement of an ICP monitor with these coagulation parameters (platelets count) safe?

- > 50.000 / mm^3^
- > 100.000 / mm^3^
- > 150.000 / mm^3^

**13.** In your daily clinical practice, do you consider the placement of an ICP monitor with these coagulation parameters **[**Prothrombin Time (PT)/ Activated Partial Thromboplastin Time (APTT)] safe?

- < 1.2 times the normal control
- < 1.5 times the normal control
- < 1.8 times the normal control

**14.** At your center, how important is ICP monitoring in patients at risk of intracranial hypertension undergoing emergency extracranial surgery*?

- Not important

- Somewhat important

- Important

- Very important

- Mandatory

**15.** In patients at risk of intracranial hypertension undergoing emergency extracranial surgery*, without ICP monitoring, do you consider systolic arterial pressure safe at a value of :

- < 70 mmHg

- 70 - 80 mmHg

- 80 - 90 mmHg

- 90 - 100 mmHg

- 100 - 110 mmHg

- > 110 mmHg

**16.** In patients at risk of intracranial hypertension undergoing emergency extracranial surgery*, without ICP monitoring, do you consider a mean arterial pressure safe at a value of :

- > 60 mmHg

- > 70 mmHg

- > 80 mmHg

- > 90 mmHg

**17.** At your center, what is the percentage of patients requiring both an emergency neurosurgical intervention (ex. hematoma evacuation) and an emergency extracranial surgical operation undergo simultaneous multisystem surgery* (intracranial +/- abdominal +/- thoracic +/- pelvic, etc.)?

**18.** At you center, do you have an established protocol to standardize emergency simultaneous multisystem surgery* (intracranial +/- abdominal +/- thoracic +/- pelvic, etc.)?

- Yes

- No

**19.** In your daily clinical practice, do you consider performing a craniotomy with these coagulation parameters (platelets count) safe?

- > 50.000 / mm^3^
- > 100.000 / mm^3^
- > 150.000 / mm^3^

**20.** In your daily clinical practice, do you consider performing a craniotomy with these coagulation parameters **[**Prothrombin Time (PT)/ Activated Partial Thromboplastin Time (APTT)] safe?

- < 1,2 times the normal control
- < 1,5 times the normal control
- < 1,8 times the normal control

**21.** How important to you is the ability to perform an emergency simultaneous multisystem surgery* (intracranial +/- abdominal +/- thoracic +/- pelvic, etc.) in the acute care setting?

- Not important

- Somewhat important

- Important

- Very important

- Mandatory

**22.** In polytrauma patients undergoing damage control resuscitation, you routinely transfuse red blood cells (RBCs)/plasma (P)/platelets (PLTs) at a ratio of:

- 1 RBCs/1 P/ 1 PLTs

- 2 RBCs/1 P/ 1 PLTs

- 3 RBCs/1 P/ 1 PLTs

- Other (please specify)

**23.** In polytrauma with TBI patients undergoing damage control resuscitation, you routinely transfuse red blood cells (RBCs)/plasma (P)/platelets (PLTs) at a ratio of:

- 1 RBCs/1 P/ 1 PLTs

- 2 RBCs/1 P/ 1 PLTs

- 3 RBCs/1 P/ 1 PLTs

- Other (please specify)

**24.** In polytrauma patients with ICP monitoring and intracranial hypertension, do you consider/monitor extracranial pressures (such as intrathoracic pressure and abdominal pressure)?

- Yes

- No

* including radiologic interventional procedures.

**Table - S1.** The countries of responders.

| **Countries**  Europe (*n* = 70) |  |
| --- | --- |
|  |  |
| Italy | 22 |
| Greece | 8 |
| Germany | 6 |
| Poland | 4 |
| France | 3 |
| Netherlands | 3 |
| Romania | 3 |
| Russia | 3 |
| UK | 3 |
| Turkey | 3 |
| Ukraine | 3 |
| Norway | 2 |
| Byelorussia | 1 |
| Bulgaria | 1 |
| Croatia | 1 |
| Georgia | 1 |
| Lithuania | 1 |
| Slovenia | 1 |
| Spain | 1 |
| Non-Europe (*n* =52)  USA | 18 |
| Brazil | 5 |
| South Africa | 4 |
| Israel | 3 |
| Australia | 3 |
| Colombia | 2 |
| India | 2 |
| Paraguay | 2 |
| Saudi Arabia | 2 |
| Argentina | 1 |
| Benin | 1 |
| Egypt | 1 |
| Jamaica | 1 |
| Japan | 1 |
| Malaysia | 1 |
| Brunei | 1 |
| Tunisia | 1 |
| United Arab Emirates | 1 |
| Panama | 1 |
| Taiwan | 1 |
|  |  |
|  |  |

Abbreviations: UK = United Kingdom, USA = United States of America.

**Table S2.** Relationship between the volume of major trauma admissions and the compliance with guidelines and the presence of protocols.

|  | Safe SBP in pts at risk of IH during EES | |  |
| --- | --- | --- | --- |
|  | < 100 mmHg | > 100 mmHg | P |
| - group A | 21 (47.7) | 23 (52.3) |  |
| - group B | 45 (57.7) | 33 (42.3) |  |
|  | | | 0.289 |
|  | Safe MAP in pts at risk of IH during EES | |  |
|  | < 80 mmHg | > 80 mmHg | P |
| - group A | 24 (54.6) | 20 (45.4) |  |
| - group B | 44 (56.4) | 34 (43.6) |  |
|  | | | 0.842 |
|  | Safe PLTs count for ICP placement | |  |
|  | < 100.000/mm^3^ | > 100.000/mm^3^ | P |
| - group A | 26 (59.1) | 18 (40.9) |  |
| - group B | 40 (51.3) | 38 (48.7) |  |
|  | | | 0.406 |
|  | Safe PLTs count for craniotomy | |  |
|  | < 100.000/mm^3^ | > 100.000/mm^3^ | P |
| - group A | 17 (38.6) | 27 (61.4) |  |
| - group B | 38 (48.7) | 40 (51.3) |  |
|  | | | 0.283 |
|  | Safe PT/aPTT for ICP placement | |  |
|  | > 1.5 | < 1.5 | P |
| - group A | 3 (6.8) | 41 (93.2) |  |
| - group B | 3 (3.9) | 75 (96.1) |  |
|  | | | 0.666 |
|  | Safe PT/aPTT for craniotomy | |  |
|  | > 1.5 | < 1.5 | P |
| - group A | 0 (0) | 44 (100) |  |
| - group B | 5 (6.4) | 73 (93.6) |  |
|  | | | 0.158 |
|  | RBCs/P/PLTs for DCR in polytrauma | |  |
|  | 2 or 1/1/1 | > 2/1/1 | P |
| - group A | 13 (29.6) | 31 (70.4) |  |
| - group B | 16 (20.5) | 62 (79.5) |  |
|  | | | 0.260 |
|  | RBCs/P/PLTs for DCR in polytrauma + TBI | |  |
|  | 2 or 1/1/1 | > 2/1/1 | P |
| - group A | 12 (27.3) | 32 (72.7) |  |
| - group B | 17 (23.8) | 61 (78.2) |  |
|  | | | 0.495 |
|  | Protocol for ICP monitoring during EES | |  |
|  | no | yes | P |
| - group A | 32 (72.7) | 12 (27.3) |  |
| - group B | 42 (53.9) | 36 (46.1) |  |
|  | | | 0.04 |
|  | Protocol for SMS | |  |
|  | no | yes | P |
| - group A | 35 (79.6) | 9 (20.4) |  |
| - group B | 54 (69.2) | 24 (30.8) |  |
|  | | | 0.218 |

P < 0.005 (Bonferroni test).

Group A = < 250 major trauma admissions/year.

Group B = > 250 major trauma admissions/year.

Abbreviations: SBP = systolic blood pressure, MAP = mean arterial pressure, ICP = intracranial pressure, IH = intracranial pressure, EES = emergency extracranial surgery PLT = platelet, PT = prothrombin time, aPTT = activated partial thromboplastin time, RBC = red blood cell, P = plasma, TBI = traumatic brain injury, SMS = simultaneous multisystem surgery.
